# Supplementary material for: Integration of Computational Pipeline to Streamline Efficacious Drug Nomination and Biomarker Discovery in Glioblastoma
Source: Cancers (Basel). 2024 Apr 28;16(9):1723. doi: 10.3390/cancers16091723 (PMC11083606; doi:10.3390/cancers16091723)
Supplement: Supplementary file 1 [file cancers-16-01723-s001.zip › cancers-2942572-supplementary.pdf]

Supplementary Materials: Integration of Computational Pipeline to Streamline Efficacious Drug Nomination and Biomarker Discovery in Glioblastoma

Table S1. Overview of the clinical and avatar datasets. Bulk gene expression derived from GBM patient and non-high grade glioma (non-HGG) data are used to computationally predict efficacious compounds against GBM. Two non-HGG patient datasets, representing low-grade glioma (LGG) from The Cancer Genome Atlas (TCGA) and Chinese Glioma Genome Atlas (CGGA), and five GBM patient datasets are used to nominate potential drugs of interest against GBM. The GBM patient datasets were obtained from TCGA, the Rembrandt study, a personal archive (CMDAT), and CGGA. The avatar dataset consists of GBM totaled 60 samples, technical replicates included, as well as 6 neural progenitor cells (NPCs).

**Non-HGG Datasets**	**GBM Patient Datasets**	**GBM Avatar Datasets**
TCGA LGG RNAseq (*n* = 516)	TCGA Microarray (*n* = 322)	Tumor spheres & tumor cells (*n* = 60)
CGGA LGG RNAseq (*n* = 282)	TCGA RNAseq (*n* = 165)	
Avatar NPCs (*n* = 6)	CGGA Microarray (*n* = 102)	
	Rembrandt Microarray (*n* = 189)	
	CMDAT Microarray (*n* = 62)	

A.

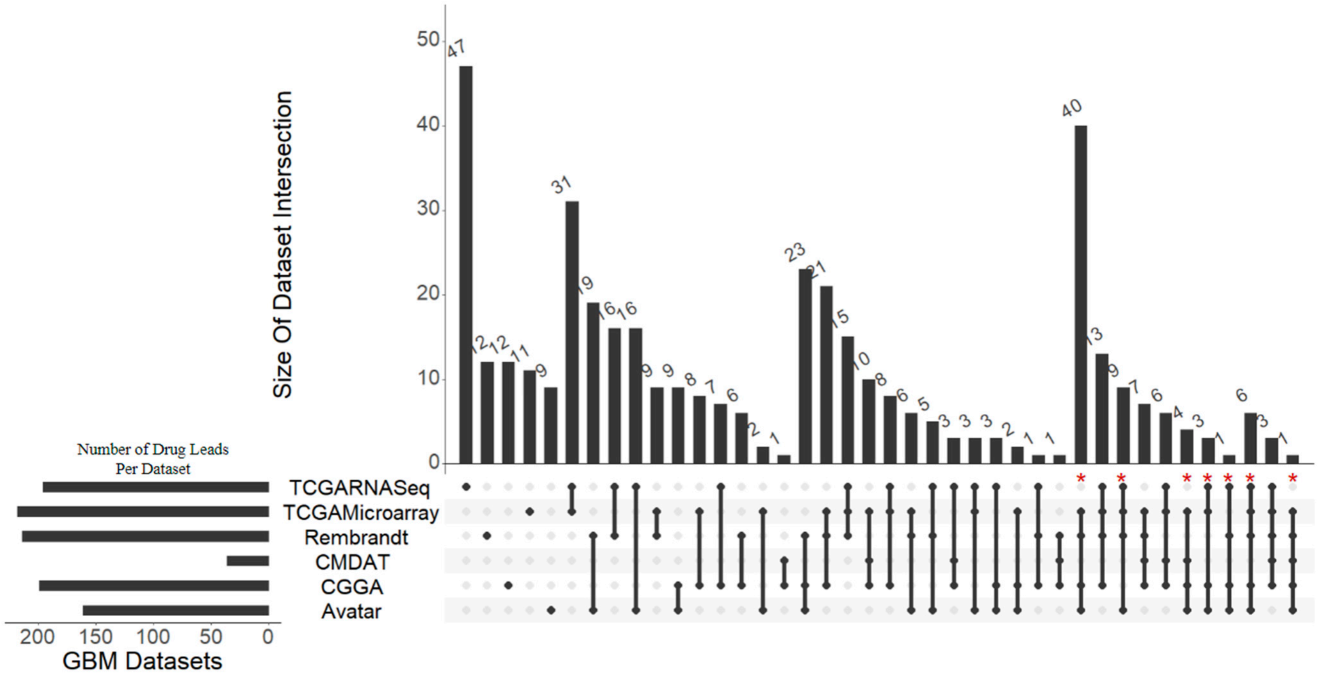

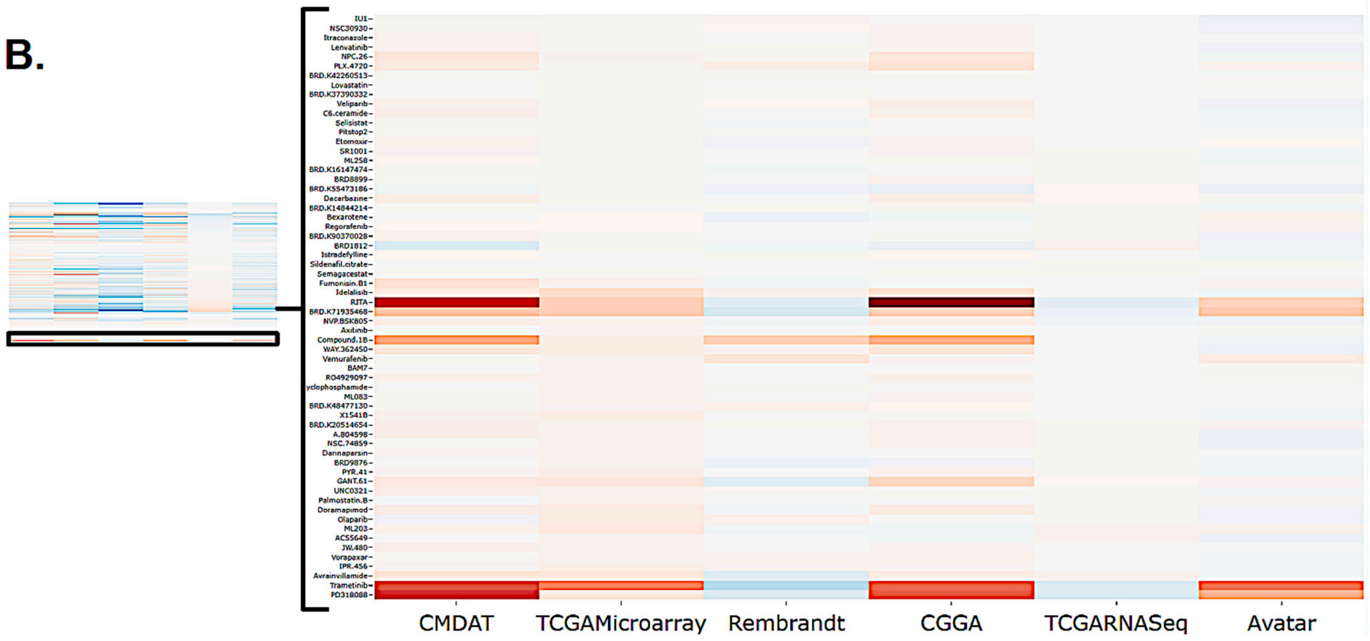

Figure S1. Drug leads identified for GBM relative to non-HGG (TCGA-LGG and NPC). A. Upset plot displaying intersections of drugs predicted to be efficacious for GBM relative to non-high grade glioma (non-HGG) across six GBM datasets. These drugs had a Hodges-Lehmann estimate (HLE) within the top 50% of drugs with a FDR corrected p-value of <= 0.05. The red asterisk indicates 62 drugs that were identified across avatar data and more than half of the clinical datasets. B. The smaller heatmap displays the standardized HLE for all significant drugs identified. The larger heatmap displays the HLE specifically for drug leads. Drugs predicted to be more efficacious for GBM are in red and include MEK inhibitor trametinib which elicited the greatest response, and those more efficacious for non-HGG are blue.

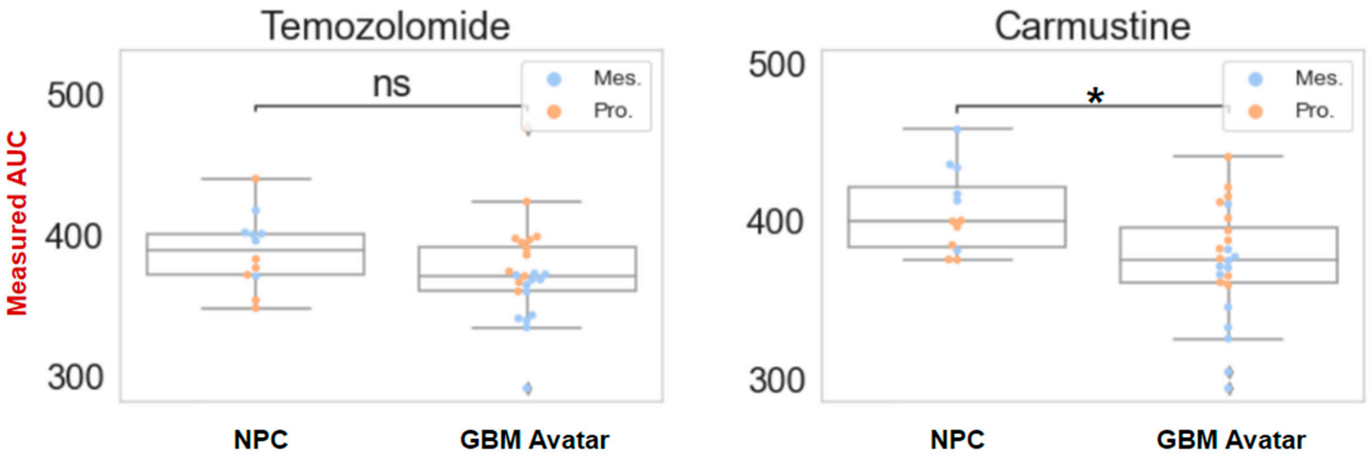

Figure S2. Standard of care agent's measured drug response across GBM mouse avatar samples. The asterisk indicates statistical significance between the NPC and GBM measured drug response. These plots display the drug response captured from experimentally testing standard of care agents temozolomide and carmustine, at various drug concentrations, in avatar mouse models. AUC (area under the dose response curve) was obtained through measuring the relative ATP scores across nine different doses, six times per concentration. Then applying the trapezoidal method to calculate the area under the dose response curve. The drug concentrations were measured by taking the natural log of the micromolar concentration. GBM samples, including the neural progenitor cell (NPC) samples that evolved to specific subtypes, are color coated.

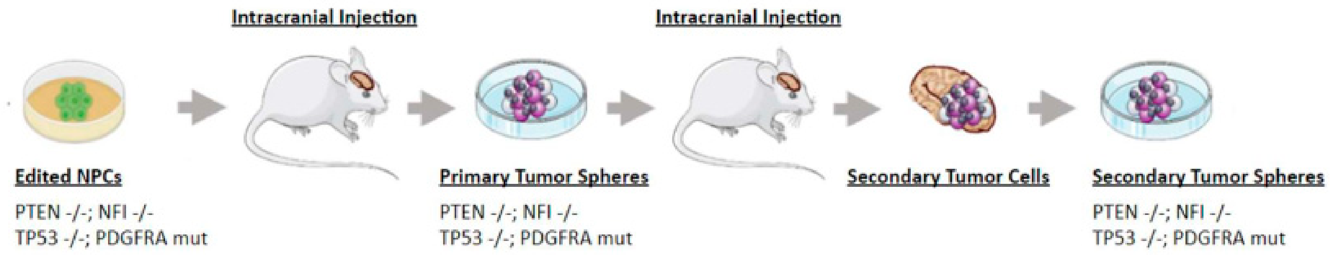

Figure S3. Generation of the mouse avatar bulk RNAsequencing data. The mouse avatar models were created following a series of steps including 1) introduction of different genetic driver mutations common in two GBM (glioblastoma) subtypes into human induced pluripotent stem cells (iPSCs) using CRISPR-Cas9. 2) differentiation of iPSCs into neural progenitor cells (NPCs), which were orthotopically engrafted into mice 3) generation of tumor cells, which were cultured to produce spheres, and this process was repeated twice 4) generation of the bulk RNAsequencing gene expression profiles of these NPCs, tumors, and spheres.

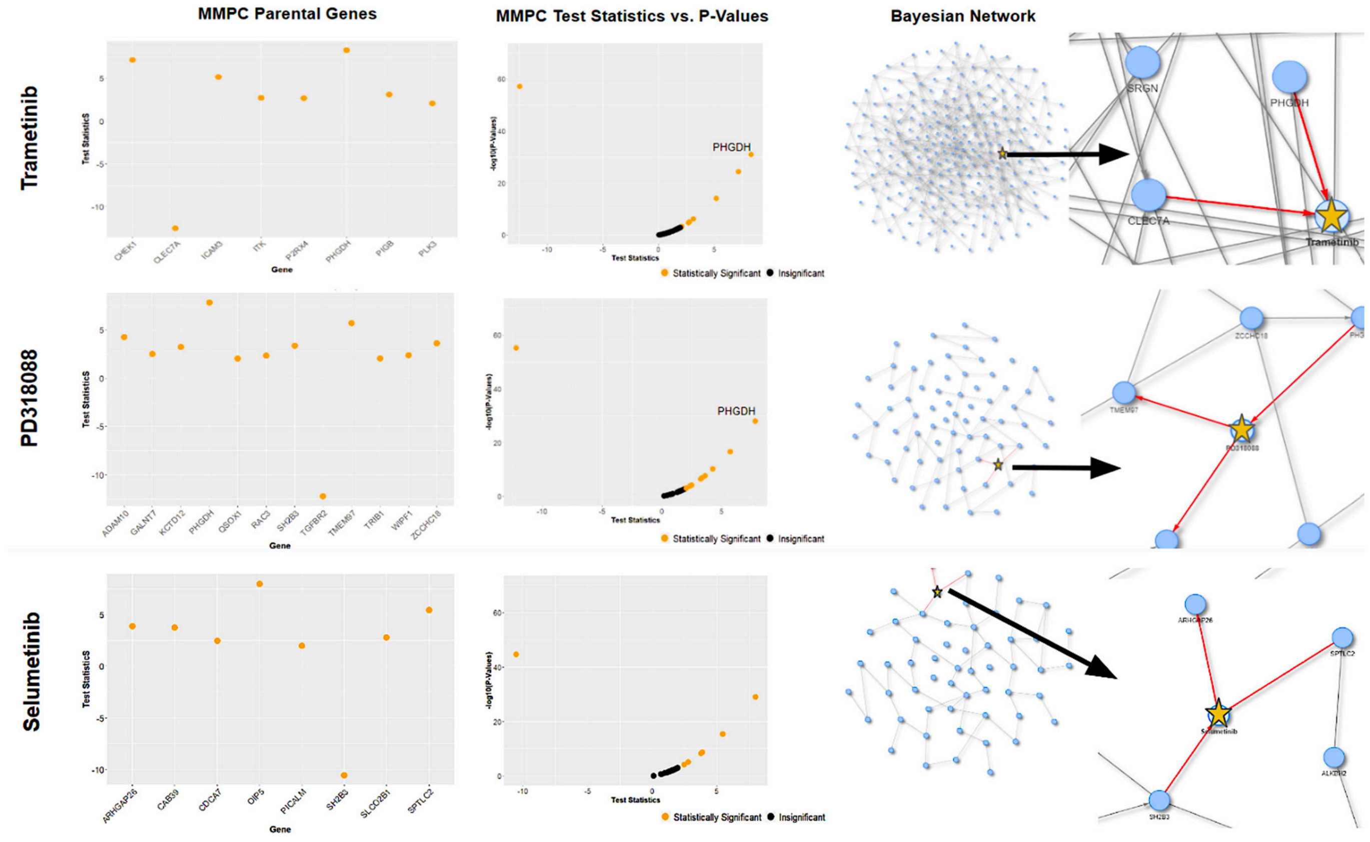

Figure S4. Multivariate method for MEK inhibitor (MEKi) biomarker discovery. MMPC parental genes were obtained through applying the Min-Max Parents Children (MMPC) algorithm to TCGA-GBM RNA sequencing data and imputed MEKi response. MMPC parental genes were indicated for each MEKi, where PHGDH was predicted to be a parental gene for both trametinib and PD31808 with the largest positive test statistic. The test statistic indicates increased MEKi sensitivity may result from PHGDH knockdown. The Bayesian network was obtained from applying the hybrid MMPC algorithm to the TCGA-GBM dataset, and PHGDH is confirmed to be a parental node to MEKi response.

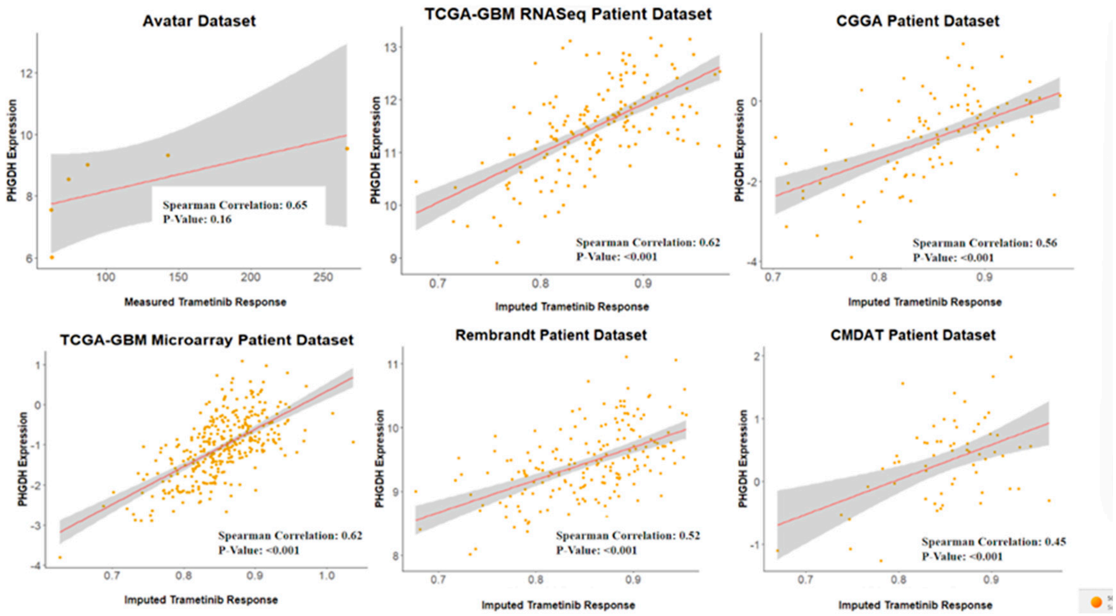

Figure S5. Linear plots displaying the relationship between PHGDH gene expression across the six GBM datasets and measured or imputed response to trametinib. The Spearman correlation coefficient and p-value between gene expression and drug response is provided. In this figure, 'Avatar' indicates the correlation between the avatar gene expression and the average area under the dose response curve measured from experimental testing.

Supplemental Data S1, S2, S3 could be accessed via this link: https://osf.io/ar9zg/. (accessed on 18 April 2024)
